# Resveratrol ameliorates the chemical and microbial induction of inflammation and insulin resistance in human placenta, adipose tissue and skeletal muscle

**DOI:** 10.1371/journal.pone.0173373

**Published:** 2017-03-09

**Authors:** Ha T. Tran, Stella Liong, Ratana Lim, Gillian Barker, Martha Lappas

**Affiliations:** 1 Obstetrics, Nutrition and Endocrinology Group, Department of Obstetrics and Gynaecology, University of Melbourne, Heidelberg, Victoria, Australia; 2 Mercy Perinatal Research Centre, Mercy Hospital for Women, Heidelberg, Victoria, Australia; Ehime University Graduate School of Medicine, JAPAN

## Abstract

Gestational diabetes mellitus (GDM), which complicates up to 20% of all pregnancies, is associated with low-grade maternal inflammation and peripheral insulin resistance. Sterile inflammation and infection are key mediators of this inflammation and peripheral insulin resistance. Resveratrol, a stilbene-type phytophenol, has been implicated to exert beneficial properties including potent anti-inflammatory and antidiabetic effects in non-pregnant humans and experimental animal models of GDM. However, studies showing the effects of resveratrol on inflammation and insulin resistance associated with GDM in human tissues have been limited. In this study, human placenta, adipose (omental and subcutaneous) tissue and skeletal muscle were stimulated with pro-inflammatory cytokines TNF-α and IL-1β, the bacterial product lipopolysaccharide (LPS) and the synthetic viral dsRNA analogue polyinosinic:polycytidylic acid (poly(I:C)) to induce a GDM-like model. Treatment with resveratrol significantly reduced the expression and secretion of pro-inflammatory cytokines IL-6, IL-1α, IL-1β and pro-inflammatory chemokines IL-8 and MCP-1 in human placenta and omental and subcutaneous adipose tissue. Resveratrol also significantly restored the defects in the insulin signalling pathway and glucose uptake induced by TNF-α, LPS and poly(I:C). Collectively, these findings suggest that resveratrol reduces inflammation and insulin resistance induced by chemical and microbial products. Resveratrol may be a useful preventative therapeutic for pregnancies complicated by inflammation and insulin resistance, like GDM.

## Introduction

Gestational diabetes mellitus (GDM) is defined as glucose intolerance of variable severity with first recognition during pregnancy [1]. The rate of GDM is increasing in parallel with the obesity epidemic, and currently affects up to 20% of pregnancies depending on the population [2, 3]. There are many short- and long-term complications associated with GDM for the mother and child [4]. In the short-term, GDM women are at a greater risk of hypertension induced by pregnancy, and even preeclampsia in severe cases [1, 5]. Hyperglycaemia during late gestation is associated with macrosomia [6]. Macrosomia is a surrogate risk for other complications, such as shoulder dystocia, induced birth and delivery by Caesarean section [6]. Babies who are not macrosomic tend to have greater adiposity [7] which predisposes them to metabolic diseases later in life, such as diabetes, cardiovascular disease (CVD) and obesity [8, 9].

Increased maternal skeletal muscle insulin resistance, a central feature of GDM pregnancies [10–14], is responsible for increased fetal nutrient supply leading to increased fetal adiposity. Increased inflammation and endotoxemia associated with GDM pregnancies [15–19] is thought to contribute to this increased maternal insulin resistance. During pregnancy, the placenta and maternal adipose tissue respond to bacterial and/or viral infections by enhancing the expression and production of pro-inflammatory mediators including the pro-inflammatory cytokines TNF-α, IL-1β, IL-1α and IL-6; and the chemokines, IL-8 and MCP-1. Increased circulating levels of these pro-inflammatory mediators can induce (i) further inflammation, (ii) and insulin resistance [15, 20–22].

Resveratrol is a stilbene-type phytophenol that is found in a wide variety of plants and fruits, such as legumes, grapes and berries. It has a wide range of beneficial properties including potent anti-inflammatory [23, 24] and antidiabetic [25–27] effects. Resveratrol reduces placental inflammation in non-human primates fed a high-fat diet (HFD) [28]. Furthermore, resveratrol improves glucose metabolism in a genetic mouse model of GDM [29]. We have previously shown that resveratrol can quench inflammation induced by bacterial endotoxin lipopolysaccharide (LPS) in human placenta [24]. Apart from this one study, little is known about the effects of resveratrol on inflammation and insulin resistance associated with GDM using human samples. Therefore, the aim of this study is to determine whether resveratrol can reduce inflammation and insulin resistance induced by inflammation and infection.

## Materials and methods

### Tissue collection

Approval for this study was obtained from the Mercy Hospital for Women’s Research and Ethics Committee and written informed consent was obtained from all participating subjects. Women were invited to provide samples on the day of admission for surgery. Women fulfilling any of the following criteria were excluded; vascular/renal complication, multiple gestations, asthma, smokers, preeclampsia, chorioamnionitis, placental abruption, acute fetal distress and women with any other adverse underlying medical conditions.

Human placenta, omental adipose tissue, and skeletal muscle (from the rectus pyramidalis) were obtained from non-obese women (body mass index, BMI <30 kg/m^2^) who delivered healthy, singleton infants at term (37–41 weeks of gestation) undergoing elective Caesarean in the absence of labour. All the tissues were delivered to the laboratory within 10 min of delivery placed in 4°C phosphate-buffered saline (PBS) ready to be processed immediately.

### Tissue explants

Human placenta, omental and subcutaneous adipose tissue and skeletal muscle were obtained from women at term elective Caesarean section. Tissues were obtained from normal glucose tolerant (NGT) women and stimulated with either bacterial lipopolysaccharide (LPS; a toll-like receptor (TLR)4 ligand), polyinosinic-polycytidylic acid (poly(I:C); a TLR3 ligand), or pro-inflammatory cytokines (i.e. IL-1β, TNF-α). Tissue explants were performed as previously described [21, 22, 24]. The tissues were blunt dissected to remove visible connective tissue, vessels and calcium-deposits then thoroughly washed with PBS. The processed tissues were pre-incubated for 1 h in Dulbecco’s Modified Eagle’s Medium (DMEM), containing 100 U/ml penicillin G, 100 μg/ml streptomycin, at 37°C in a humidified incubator of 5% CO_2_ and 8% O_2_ (for placenta) and 21% O_2_ (for skeletal muscle and adipose tissue). The samples were then blotted dry on filter paper; 100 mg wet weight (for placenta and adipose) or 50 mg wet weight (for skeletal muscle) per well was transferred to a 24-well tissue culture plate and incubated in 1 ml DMEM for 20 h. To determine the effects of resveratrol on placental, omental and subcutaneous adipose tissue and skeletal muscle, these tissues were incubated in 10 μg/ml LPS, 50 μg/ml poly(I:C), 10 ng/ml TNF-α, 5 ng/ml IL-1β with or without 200 μM resveratrol (AdooQ BioScience, Irvine, CA, USA). The optimised concentration of resveratrol [24] and the inflammatory mediators [20, 24, 30] were determined by previously published studies. After final incubation, tissue and media were collected separately and stored at -80°C for further analysis as detailed below. Each treatment was performed on tissues obtained from six patients. Experiments were performed in duplicate; the average of the duplicate was used for final data analysis.

To assess the effects of resveratrol on the insulin signalling pathway in skeletal muscle, explants were performed as detailed above. However, after 20 h incubation, tissues were incubated with 0.1 μM insulin for 30 min to activate the insulin signalling pathway. After final incubation, tissue was collected and assessment of glucose uptake and expression of the insulin signalling proteins by Western blot are detailed below. Each treatment was performed on tissues obtained from six patients for both the glucose uptake assays and Western blotting.

### RNA extraction and quantitative RT-PCR (qRT-PCR)

Total RNA was extracted from tissues using TRIsure reagent according to manufacturer’s instructions (Bioline, Alexandria, NSW, Australia), as previously described [30]. RNA concentration and purity were determined using a NanoDrop ND1000 spectrophotometer (Thermo Scientific, Pittsburgh, PA). RNA was converted to cDNA using the SuperScript® VILO™ cDNA Synthesis Kit (Thermo Fisher Scientific; Scoresby, Vic, Australia) according to the manufacturer’s instructions. The cDNA was diluted fifty-fold, and 4 μl of this was used to perform qRT-PCR using SensiFAST™ SYBR No-ROX Kit (Bioline, Alexandria, NSW, Australia) and pre-designed and validated QuantiTect primers (Qiagen; Chadstone Centre, Vic, Australia). The RT-PCR was performed using a CFX384 Real-Time PCR detection system from Bio-Rad Laboratories (Hercules, California, USA). Average gene Ct values were normalised against two housekeeping genes (β2-Microglobulin (B2M) and 18S rRNA). Of note, there was no effect of experimental treatment on B2M mRNA or 18S rRNA expression. Fold differences were determined using the comparative Ct method.

### Western blotting

Western blotting was performed as previously described [31]. Blots were cut into three sections of MW ranges: 250–100 kD, 100–75 kD and 75–37 kD. The 250–100 kD section was probed with 1 μg/ml rabbit polyclonal phosphorylated (Tyr1229) IRS-1 (sc-17202; Santa Cruz Biotechnology; Santa Cruz, CA, USA); the 100–75 kD section was probed with 1 μg/ml rabbit polyclonal phosphorylated (Tyr 1162/1163) IR-β (sc-25103; Santa Cruz Biotechnology, Santa Cruz, CA, USA); and the 75–37 kD section was probed with 1 μg/ml rabbit polyclonal GLUT-4 (SAB4300667; Sigma-Aldrich; St. Louis, MO, USA). Antibodies were incubated in blocking buffer (5% BSA in TBS with 0.05% Tween-20) for 16 h at 4°C. Membranes were viewed and analysed as described above. For normalisation, blots were stripped and re-probed with either1 μg/ml rabbit polyclonal IR-β (sc-711; Santa Cruz Biotechnology, Santa Cruz, CA, USA), 1 μg/ml rabbit polyclonal IRS-1 (sc-560; Santa Cruz Biotechnology; Santa Cruz, CA, USA) or β-actin (1:20,000; A5316; Sigma-Aldrich; St. Louis, MO, USA). Phosphorylated IRS-1 and IR-β data were corrected for background and normalised to total IRS-1 and IR-β, respectively. GLUT-4 data was corrected for background and normalised to β-actin (1:20,000; A5316; Sigma-Aldrich; St. Louis, MO, USA). The blots were treated with HRP-conjugated secondary antibody (1:2500) obtained from Santa Cruz Biotechnology for 45 mins at room temperature. The specific signals were visualised using Western blotting luminol reagent (Santa Cruz Biotechnology, Santa Cruz, CA, USA). Membranes were viewed and analysed using the XRS ChemiDoc system (Bio-Rad Laboratories; Gladesville, NSW, Australia). Semi-quantitative analysis of the relative density of the bands in Western blots was performed using Image Lab 3.0 (Bio-Rad Laboratories; Gladesville, NSW, Australia).

### Enzyme immunoassays

The release of IL-6, IL-8 and MCP-1 into the incubation medium was performed by sandwich ELISA according to the manufacturer’s instructions (Life Technologies; Mulgrave, Vic, Australia). All data were corrected for total protein and expressed as either pg or ng per mg protein. The protein content of tissue homogenates was determined using BCA protein assay (Thermo Fisher Scientific; Scoresby, Vic, Australia), using BSA as a reference standard, as previously described [32]. The calculated interassay and intraassay coefficients of variation (CV) were all less than 10%.

### Glucose uptake

Skeletal muscle explants were performed as detailed above and glucose uptake was performed as previously described [31]. Briefly, after final incubation with treatment, tissues were pre-incubated in the absence or presence of 20 μM cytochalasin B in Krebs buffer for 5 mins. 2-Deoxy-D-glucose (2DG) uptake was measured by adding 3 μCi/ml [^14^C]-2DG (Perkin Elmer) and 1 mM 2DG to Krebs buffer containing 0.5% BSA (fatty acid free) and 0.1 μM insulin for 20 min. Tissues were then collected and washed three times in PBS, blotted dry on filter paper and then solubilised for 4 h in 0.5 ml 1 M NaOH at 60°C. Tissues were neutralised with 0.5 ml 1 M HCl and then centrifuged at 15,000 g for 5 min to pellet insoluble material. The supernatant was transferred to a vial containing 3 ml of liquid scintillation fluid. All samples were counted for radioactivity in a liquid scintillation counter. GLUT-specific glucose uptake was measured by subtracting values for [^14^C]-2DG uptake in the presence of 20 μM cytochalasin B. Glucose uptake was performed on tissues obtained from six patients.

### Statistical analysis

Statistics was performed on the normalised data unless otherwise specified. All statistical analyses were undertaken using GraphPad Prism (GraphPad Software, La Jolla, CA, USA). The homogeneity of data was assessed by the Bartlett’s test. For non-parametric data, the Friedman test was used while parametric data were assessed by a one-way ANOVA using Fisher's Least Significant Difference (LSD) post hoc test to allow multiple comparisons between the groups. Statistical significance was ascribed to a *P* value <0.05. Data were expressed as mean ± SEM.

## Results

### Effect of resveratrol on inflammation in placenta

**Fig 1:**
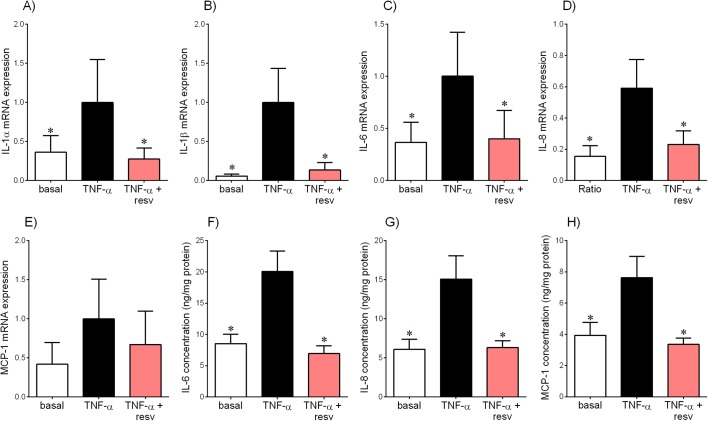
Effect of resveratrol on TNF-α-induced pro-inflammatory cytokines and chemokines in placenta. Human placenta was incubated with 10 ng/ml TNF-α in the absence or presence of 200 μM resveratrol (resv) for 20 h (n = 6 patients). **(A-E)** IL-1α, IL-1β, IL-6, IL-8 and MCP-1 mRNA expression was analysed by qRT-PCR and the fold change was calculated relative to TNF-α. **(F-H)** The incubation medium was assayed for concentration of IL-6, IL-8 and MCP-1 release by ELISA. All data are displayed as mean ± SEM. *P<0.05 vs. TNF-α.

**Fig 2:**
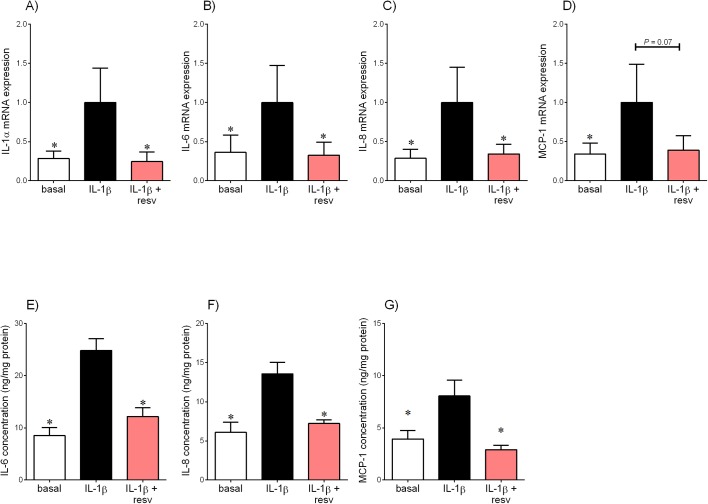
Effect of resveratrol on IL-1β-induced pro-inflammatory cytokines and chemokines in placenta. Human placenta was incubated with 5 ng/ml IL-1β in the absence or presence of 200 μM resveratrol (resv) for 20 h (n = 6 patients). **(A-D)** IL-1α, IL-6, IL-8 and MCP-1 mRNA expression was analysed by qRT-PCR and the fold change was calculated relative to IL-1β. **(E-G)** The incubation medium was assayed for concentration of IL-6, IL-8 and MCP-1 release by ELISA. All data are displayed as mean ± SEM. *P<0.05 vs. IL-1β.

**Fig 3:**
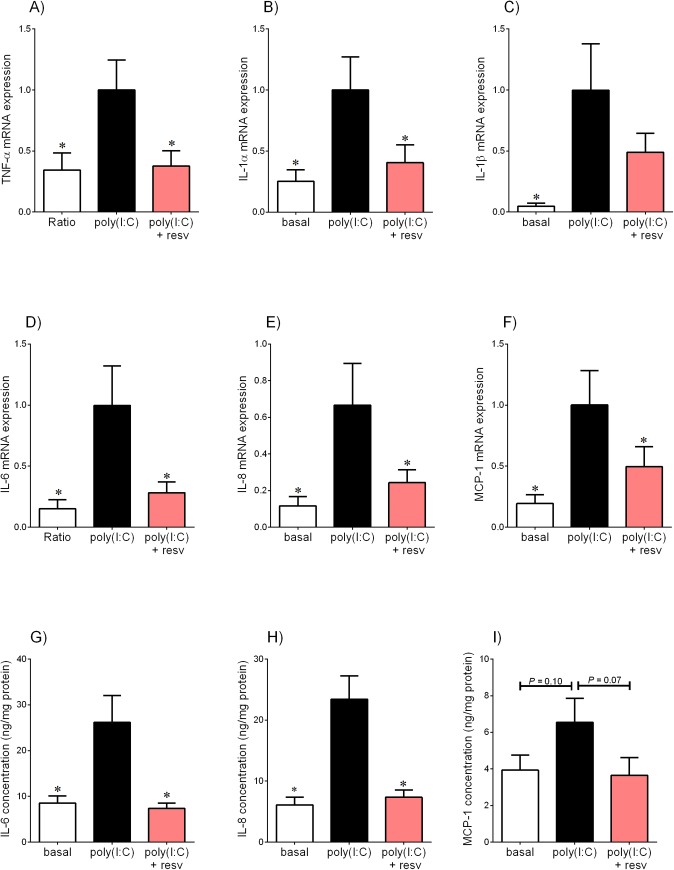
Effect of resveratrol on poly(I:C)-induced pro-inflammatory cytokines and chemokines in placenta. Human placenta was incubated with 50 μg/ml poly(I:C) in the absence or presence of 200 μM resveratrol (resv) for 20 h (n = 6 patients). **(A-F)** TNF-α, IL-1α, IL-1β, IL-6, IL-8 and MCP-1 mRNA expression was analysed by qRT-PCR and the fold change was calculated relative to poly(I:C). **(G-I)** The incubation medium was assayed for concentration of IL-6, IL-8 and MCP-1 release by ELISA. All data are displayed as mean ± SEM. *P<0.05 vs. poly(I:C).

Human placenta was incubated with resveratrol, in the presence of TNF-α (Fig 1), IL-1β (Fig 2) or poly(I:C) (Fig 3). As demonstrated in Fig 1, TNF-α treatment significantly increased IL-1α, IL-1β, IL-6 and IL-8 mRNA expression (Fig 1A–1D) and release of IL-6, IL-8 and MCP-1 (Fig 1F–1H). There was no effect of TNF-α on MCP-1 mRNA expression (Fig 1E). Treatment with resveratrol significantly attenuated TNF-α-stimulated IL-1α, IL-1β, IL-6 and IL-8 mRNA expression and release of IL-6, IL-8 and MCP-1.

Fig 2 depicts the effect of resveratrol on IL-1β-stimulated pro-inflammatory cytokine and chemokine expression in human placenta. As expected, treatment with IL-1β significantly increased IL-1α, IL-6, IL-8 and MCP-1 mRNA expression (Fig 2A–2D) and release of IL-6, IL-8 and MCP-1 (Fig 2E–2G). Treatment with resveratrol significantly attenuated IL-1β-stimulated IL-1α, IL-6 and IL-8 mRNA expression and release of IL-6, IL-8 and MCP-1. IL-1β-induced MCP-1 mRNA expression was decreased by resveratrol; however, this failed to reach statistical significance (*P* = 0.07; Fig 2D).

The effect of resveratrol on poly(I:C)-induced cytokine and chemokine expression is demonstrated in Fig 3. Treatment with poly(I:C) significantly increased TNF-α, IL-1α, IL-1β, IL-6, IL-8 and MCP-1 mRNA expression (Fig 3A–3F) and release of IL-6 and IL-8 (Fig 3G and 3H). Treatment with resveratrol significantly attenuated poly(I:C)-induced expression of TNF-α, IL-1α, IL-1β, IL-6, IL-8 and MCP-1 mRNA expression and release of IL-6 and IL-8. Resveratrol decreased poly(I:C)-induced IL-1β mRNA expression (Fig 3C) and MCP-1 release, however this was not statistically significant (Fig 3I).

### Effect of resveratrol on inflammation in omental adipose tissue

**Fig 4:**
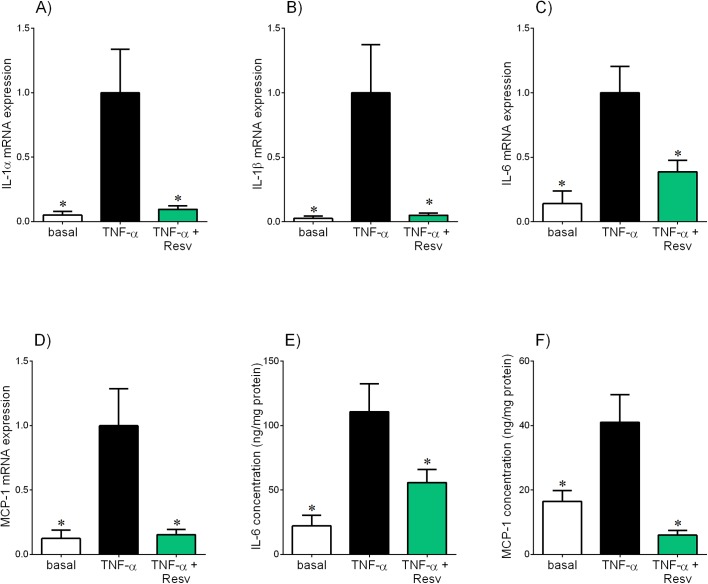
Effect of resveratrol on TNF-α-induced pro-inflammatory cytokines and chemokines in omental adipose tissue. Human omental adipose tissue was incubated with 10 ng/ml TNF-α in the absence or presence of 200 μM resveratrol (resv) for 20 h (n = 6 patients). **(A-D)** IL-1α, IL-1β, IL-6 and MCP-1 mRNA expression was analysed by qRT-PCR and the fold change was calculated relative to TNF-α. **(E,F)** The incubation medium was assayed for concentration of IL-6 and MCP-1 release by ELISA. All data are displayed as mean ± SEM. *P<0.05 vs. TNF-α.

**Fig 5:**
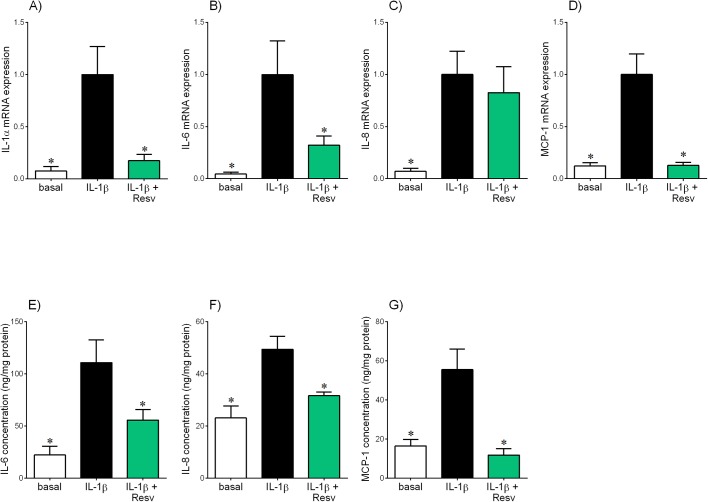
Effect of resveratrol on IL-1β-induced pro-inflammatory cytokines and chemokines in in omental adipose tissue. Human omental adipose tissue was incubated with 5 ng/ml IL-1β in the absence or presence of 200 μM resveratrol (resv) for 20 h (n = 6 patients). **(A-D)** IL-1α, IL-6, IL-8 and MCP-1 mRNA expression was analysed by qRT-PCR and the fold change was calculated relative to IL-1β. **(E-G)** The incubation medium was assayed for concentration of IL-6, IL-8 and MCP-1 release by ELISA. All data are displayed as mean ± SEM. *P<0.05 vs. IL-1β.

**Fig 6:**
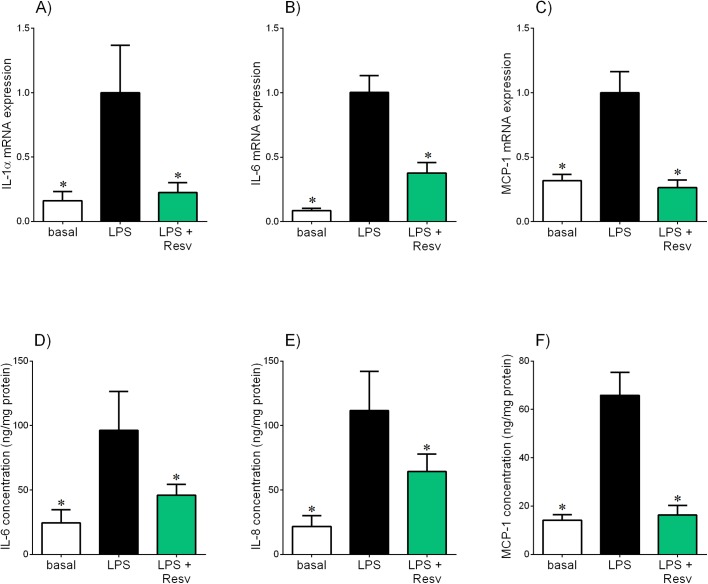
Effect of resveratrol on LPS-induced pro-inflammatory cytokines and chemokines in in omental adipose tissue. Human omental adipose tissue was incubated with 10 μg/ml LPS in the absence or presence of 200 μM resveratrol (resv) for 20 h (n = 6 patients). **(A-C)** IL-1α, IL-6 and MCP-1 mRNA expression was analysed by qRT-PCR and the fold change was calculated relative to LPS. **(D-F)** The incubation medium was assayed for concentration of IL-6, IL-8 and MCP-1 release by ELISA. All data are displayed as mean ± SEM. *P<0.05 vs. LPS.

The effect of resveratrol treatment in omental adipose tissue was also studied, and is depicted in Figs 4–6. As show in Fig 4, treatment with TNF-α significantly increased IL-1α, IL-1β, IL-6 and MCP-1 mRNA expression and release of IL-6 and MCP-1 in omental tissue. Resveratrol treatment significantly decreased TNF-α-stimulated IL-1α, IL-1β, IL-6 and MCP-1 mRNA expression and release of IL-6 and MCP-1.

The effect of resveratrol treatment in omental tissue in the presence of IL-1β is shown in Fig 5. As expected, IL-1β treatment significantly increased IL-1α, IL-6, IL-8 and MCP-1 mRNA expression and release of IL-6, IL-8 and MCP-1. A significant reduction in IL-1β-stimulated IL-1α, IL-6 and MCP-1 mRNA expression and release of IL-6, IL-8 and MCP-1 was observed in omental tissue co-treated with resveratrol. There was, however, no effect of resveratrol on IL-1β-induced IL-8 mRNA expression (Fig 5C).

The effect of resveratrol on the production of pro-inflammatory cytokines and chemokines was also determined in omental tissue treated with LPS, as shown in Fig 6. As expected, treatment with LPS significantly increased mRNA expression of IL-1α, IL-6 and MCP-1 and release of IL-6, IL-8 and MCP-1. Resveratrol treatment significantly attenuated LPS-stimulated IL-1α, IL-6 and MCP-1 mRNA expression and release of IL-6, IL-8 and MCP-1.

Similar results were obtained from subcutaneous adipose tissue (S1 and S2 Figs), whereby resveratrol treatment attenuates IL-1β and LPS-stimulated pro-inflammatory cytokine and chemokine expression.

### Effect of resveratrol on skeletal muscle insulin signalling

**Fig 7:**
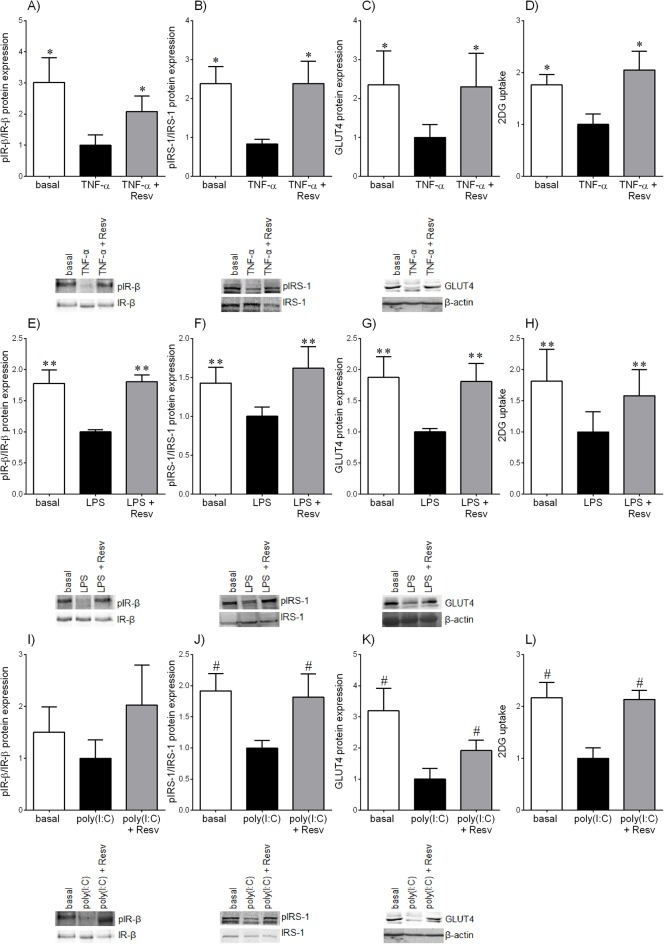
Effect of resveratrol on the insulin signalling pathway. Human skeletal muscle tissue was incubated with **(A-D)** 10 ng/ml TNF-α, **(E-H)** 10 μg/ml LPS or **(I-L)** 50 μg/ml poly(I:C) for 20 h with or without 200 μM resveratrol (resv), followed by a 30 min incubation with 0.1 μM insulin (n = 6 patients). **(A,E,I)** Phosphorylated IR-β (pIR-β) protein expression was normalised to total IR-β protein expression. A representative Western blot from 1 patient is also shown. **(B,F,J)** Tyrosine phosphorylated IRS-1 (pIRS-1) protein expression was normalised to total IRS-1 protein expression. A representative Western blot from 1 patient is also shown. **(C,G,K)** GLUT4 protein expression was normalised to β-actin protein expression. A representative Western blot from 1 patient is also shown. **(D,H,L)** Glucose uptake was analysed using a radiolabelled 2DG uptake assay. For all data, the fold change was calculated relative to TNF-α, LPS or poly(I:C) and data are presented as mean ± SEM. *P<0.05 vs. TNF-α; **P<0.05 vs. LPS; #*P*<0.05 vs. poly(I:C).

The effect of resveratrol on insulin signalling was determined in skeletal muscle in the presence of TNF-α, LPS or poly(I:C). Both TNF-α and LPS treatments significantly attenuated phosphorylated IR-β protein expression (Fig 7A and 7E). Co-incubation with resveratrol restored phosphorylated IR-β protein expression to basal levels. While a similar result was obtained with resveratrol treatment in the presence of poly(I:C) (Fig 7I), the differences did not reach statistical significance. Treatment of skeletal muscle with TNF-α, LPS and poly(I:C) significantly attenuated phosphorylated IRS-1 protein expression (Fig 7B, 7F and 7J), GLUT4 protein expression (Fig 7C, 7G and 7K) and 2DG uptake (Fig 7D, 7H and 7L). Co-incubation with resveratrol in skeletal muscle treated with either TNF-α, LPS or poly(I:C), significantly restored phosphorylated IRS-1 protein expression (Fig 7B, 7F and 7J), GLUT4 protein expression (Fig 7C, 7G and 7K) and 2DG uptake (Fig 7D, 7H and 7L) to basal levels.

## Discussion

This study has demonstrated that the phytophenol resveratrol can significantly decrease chemical and microbial induction of inflammation in human placenta and adipose tissues obtained from pregnant women. Resveratrol was also able to significantly restore the impaired insulin signalling pathway and insulin-mediated glucose uptake in human skeletal muscle obtained from pregnant women.

Low-grade maternal inflammation is a key feature of pregnancies complicated by GDM [33–39]. There is extensive evidence that show human placenta and adipose tissue are important sites involved in the propagation of this maternal inflammation [15, 21, 22, 30, 32, 40–48]. In this study, human placenta and adipose tissue (omental and subcutaneous) were stimulated with the pro-inflammatory cytokines TNF-α and IL-1β in order to induce an inflammatory state. Both TNF-α and IL-1β significantly induced the production of pro-inflammatory cytokines TNF-α, IL-1α, IL-1β, and IL-6, and chemokines, IL-8 and MCP-1 in placenta, and omental and subcutaneous adipose tissue obtained from pregnant women. Co-incubation with resveratrol, however, significantly reduced TNF-α- and IL-1β-induced inflammation in placenta and adipose tissue.

Recently, there has been some evidence to suggest that bacterial and viral infection are associated with GDM [20, 49–52]. It is not known whether infection contributes to the pathophysiology of GDM; however, the placenta and adipose tissue produce can secrete pro-inflammatory mediators in response to pathogens and/or their by products. For example, studies have demonstrated the bacterial endotoxin LPS [24, 32, 47], and viral dsRNA analogue poly(I:C) [21, 53] can induce the expression of pro-inflammatory cytokines in placenta and adipose tissue *in vitro*. We have previously shown resveratrol to significantly attenuate LPS-induced inflammation in human placenta [24]. Here, we extend these studies and demonstrate resveratrol also significantly reduces mRNA expression and secretion of pro-inflammatory cytokines (IL-1α, IL-1β and IL-6) chemokines (IL-8 and MCP-1) in human placenta and adipose tissue (omental and subcutaneous) stimulated by poly(I:C) or pro-inflammatory cytokines IL-1β and TNF-α.

In corroboration with our findings, experimental animal studies have also demonstrated that resveratrol can ameliorate HFD-induced inflammation during pregnancy. Specifically, resveratrol decreased HFD-induced IL-1β and RANTES (regulated on activation, normal T-cell expressed and secreted) in the placenta of non-human primates [28]; and in mice, resveratrol also decreased HFD-induced serum levels of TNF-α and MCP-1 and mRNA expression of TNF-α, IL-6, IFN-α and IFN-β in adipose tissue [54]. However, it should be noted that these animal studies on resveratrol use nutritional manipulation (i.e. HFD) to induce a model of GDM. Although some women develop GDM due to pre-existing obesity, many women who present with GDM are lean pre-pregnancy. Moreover, these HFD-induced models of GDM do not account for other factors such as genetic susceptibility on the development of GDM.

Increased maternal peripheral insulin resistance is another central feature in the pathophysiology of GDM [9, 10, 12, 55]. Low-grade maternal inflammation induced by sterile inflammation or an underlying maternal infection can contribute to the development of peripheral insulin resistance in skeletal muscle of pregnant women [5, 10, 55–57]. Studies have shown that the insulin signalling pathway and glucose uptake in skeletal muscle from pregnant women are significantly impaired by pro-inflammatory cytokines TNF-α and IL-1β, and also by LPS and poly(I:C) [20, 22, 31]. Thus, in order to test the effect of resveratrol on the insulin signalling pathway, human skeletal muscle were stimulated by TNF-α, LPS or poly(I:C) in order to generate a model of insulin resistance. Excitingly, resveratrol was able to restore the insulin signalling pathway and glucose uptake to basal levels in skeletal muscle impaired by TNF-α, LPS and poly(I:C) treatments. In agreement with our findings, similar beneficial effects on insulin resistance and glucose metabolism by resveratrol have also been demonstrated in experimental pregnant animal models. For example, resveratrol improved glucose metabolism and insulin tolerance in a particular genetic strain of mice that spontaneously develop GDM when pregnant [29]. Studies conducted on pregnant non-human primates consuming a Western-style diet have also shown improvements in their glucose tolerance and decreases in placental inflammation and fetal liver triglyceride deposition when supplementing their diets with resveratrol throughout pregnancy [28]. However, this study also found resveratrol to alter fetal pancreatic development including increased pancreatic mass and exocrine cell proliferation.

The effects of resveratrol on glucose metabolism in humans have been studied, however, only in short-term non-pregnant human clinical trials. Nonetheless, these studies have shown that resveratrol can improve insulin sensitivity and glucose metabolism. In a study performed with 19 diabetic Hungarian patients, those who received resveratrol for 4 weeks showed improved insulin sensitivity [27]. In another study, 11 obese middle-aged Dutch men received 0.15 g resveratrol/day for a month subsequently demonstrated reduced circulating plasma glucose and insulin levels, however only displayed a modest improvement in their insulin sensitivity [58]. This was confirmed in a larger double-blinded cohort study consisting of 66 type 2 diabetes patients in Iran, in which 1 g resveratrol/day was administered for 6 weeks [59]. Although these human studies show significant promise regarding the beneficial effects of resveratrol in alleviating insulin resistance and improving glucose metabolism, there is a lack of research in the use of resveratrol supplementation by pregnant women with GDM; this presents a new and important area of study, not only on maternal, but also fetal consequences.

The health risks associated with pregnancies complicated by GDM are not only restricted to mothers but can have devastating and long-term effects on the developing fetus. Studies have shown that placental inflammation can alter maternal-fetal nutrient transportation. For example, IL-6 has been demonstrated to stimulate fatty acid (FA) accumulation [60] and System A amino acid transporter activity and expression in primary human trophoblast cells [61]. In adipose tissue obtained from women with GDM, TNF-α, IL-1β and leptin expression levels are increased compared to normal healthy women [41]. This increase in TNF-α, IL-1β and leptin expression is subsequently associated with decreased expression of genes involved in FA uptake and transport, such as the lipoprotein lipase (LPL) and FATP (FA transport proteins)-2 and -6 [41]. Thus, these alterations in the nutrient transportation system may further exacerbate fatty acid accumulation in the placenta. Overall, these findings suggest that pro-inflammatory cytokines may contribute to the development of fetal macrosomia and increase fetal adiposity [62], which markedly increases the risk of obesity [8] and metabolic disease [9, 63] later in life for the offspring from pregnancies complicated by GDM. A recent study on pregnant Japanese macaques fed a HFD demonstrated that resveratrol increased docosahexaenoic acid (DHA) uptake capacity [64], which may be due to improvements in uterine and umbilical blood flow by resveratrol [28]. DHA is a long-chain polyunsaturated FA, which is critical for fetal neurological and cardiovascular development [65]. Although these animal studies have found resveratrol to exert beneficial effects on maternal glucose metabolism and insulin sensitivity, there has been some concerning data on the effect of resveratrol supplementation on fetal pancreatic development [28]. Thus, more extensive studies are warranted in order to assess the long-term effects of resveratrol supplementation on offspring development.

A limitation of this study is the use of TNF-α, IL-1β, LPS or poly(I:C) to generate a GDM-like environment in placenta, adipose tissue and skeletal muscle obtained from normal glucose tolerant pregnant women. Thus, the findings of this study should be interpreted with some caution. It should be noted that the aim of this study was to assess whether resveratrol was able to prevent the development of GDM caused by chronic low-grade inflammation and increased peripheral insulin resistance. Many women diagnosed with GDM will already be undertaking insulin therapy to manage their diabetes, and thus would not only act as confounders to our data, but it would be difficult to assess the role of resveratrol as a preventative for the development of GDM. Notwithstanding these limitations, inflammation and infection are central to the pathophysiology of GDM and/or obesity [16, 17, 40, 55, 66–68]. As a result, our findings that show resveratrol can decrease inflammation in placenta and adipose tissue and increase insulin sensitivity in skeletal muscle induced by infection and sterile inflammation remains to be of particular interest as a potential therapeutic in the prevention or management of GDM and/or obesity.

The consequences of GDM are not restricted to pregnancy but are strongly associated with adverse perinatal morbidity, including long-term health risks such as obesity and development of type 2 diabetes later in life [8]. Current therapeutics for GDM are restricted in only managing maternal hyperglycaemia with no effect on reducing inflammation that is associated with GDM. Inflammation plays a key role in GDM pathophysiology by inducing peripheral insulin resistance. Studies have implicated the placenta and adipose tissue to be critical sites in propagating this increased maternal inflammation associated with GDM. To our knowledge, this is the first study to show that resveratrol can decrease inflammation in human placenta and adipose tissue obtained from pregnant women and improve skeletal muscle insulin resistance *in vitro*. Experimental animal models have shown resveratrol to exert anti-inflammatory and antidiabetic effects *in vivo* [28, 29, 54, 64, 69]. Given that clinical trials have shown resveratrol to be safe for human consumption [27, 58, 59, 70, 71] and a cheap, commercially available preparation of resveratrol exists, human clinical trials to assess the effect of resveratrol as a preventative for GDM are highly feasible.

## Supporting information

S1 Fig Effect of resveratrol on IL-1β-induced pro-inflammatory cytokines and chemokines in subcutaneous adipose tissue. (DOCX)

S2 Fig Effect of resveratrol on LPS-induced pro-inflammatory cytokines and chemokines in in subcutaneous adipose tissue. (DOCX)
