# Supplementary material for: Resveratrol ameliorates the chemical and microbial induction of inflammation and insulin resistance in human placenta, adipose tissue and skeletal muscle
Source: PLoS One. 2017 Mar 9;12(3):e0173373. doi: 10.1371/journal.pone.0173373 (PMC5344491; doi:10.1371/journal.pone.0173373)
Supplement: S2 Fig — (DOCX) [file pone.0173373.s002.docx]

**

**

**S2 Fig. Effect of resveratrol on LPS-induced pro-inflammatory cytokines and chemokines in in subcutaneous adipose tissue.**

Human subcutaneous adipose tissue was incubated with 10 μg/ml LPS in the absence or presence of 200 µM resveratrol (resv) for 20 h (n=6 patients). **(A-C)** IL-1α, IL-6 and MCP-1 mRNA expression was analysed by qRT-PCR and the fold change was calculated relative to LPS. **(D-F)** The incubation medium was assayed for concentration of IL-6, IL-8 and MCP-1 release by ELISA. All data are displayed as mean ± SEM. **P*<0.05 vs. LPS.
